# Navigating Potential Dengue and Its Mimics in a Febrile Returning Traveller: A Case Report

**DOI:** 10.7759/cureus.101418

**Published:** 2026-01-13

**Authors:** Chloé Moran

**Affiliations:** 1 Emergency Medicine, Manchester Royal Infirmary, Manchester, GBR

**Keywords:** dengue, febrile, fever, traveller, tropical disease, tropical infection

## Abstract

Fever is a common symptom in returning travellers. This prompts healthcare providers to consider and investigate potentially serious infections requiring timely diagnosis and management. The following case describes a traveller returning from Bangladesh presenting with symptoms indicative of dengue. These include fever, rash, nausea, vomiting, myalgia and conjunctivitis. However, his symptoms defied the expected pattern of presentation, posing a diagnostic challenge. No laboratory confirmation of prior dengue infection was available. He was discharged as having a viral fever with a rash and the possibility that this was a secondary infection occurring after dengue. The case highlights the complexity of febrile travellers. It also reviews the literature on dengue, offering important learning opportunities for clinicians encountering comparable presentations. The case emphasises the need for a balanced diagnostic approach in thoroughly ruling out serious tropical infections without neglecting common community-acquired viruses.

## Introduction

Fever in the returning traveller represents a significant proportion of acute presentations to emergency departments worldwide [1]. The surge in travel in the last decade has exposed populations to a wider range of global infections [2]. In 2024, 904 dengue cases were reported from returning travellers to the UK, alongside 753 malaria cases [3,4]. These figures highlight the public health relevance of recognising tropical infections and the diagnostic challenges they present in non-endemic settings. Investigating tropical infections in returning travellers requires appropriate diagnostic procedures, careful management, and appropriate infection control measures [5]. Such measures are justified and prudent, given the potential health risks and public health implications associated with these conditions [5]. 

However, non-endemic countries face the challenge of differentiating tropical infections from other common illnesses [6]. Self-limiting infections, such as Influenza A and B, Rhinovirus, Adenovirus and Epstein-Barr Virus, must also not be forgotten [7]. They are frequent pathogens in fever in returning travellers, even those returning from high-risk countries [8]. This is further complicated by increasing global travel, as well as climate change and urbanisation, which contribute to the spread of vector-borne diseases [9]. 

This case highlights the public health considerations necessary to reduce misdiagnosis, transmission, and the burden of tropical infections. Clinicians must think beyond their local infection-risks and consider the broader context of global disease patterns [10]. This is particularly important in large metropolitan cities; major airports serve as key points of international connectivity [11]. Clinicians must consequently be aware of tropical infections such as dengue, which was a primary concern in this case. 

## Case presentation

A 51-year-old male patient presented to the emergency department one week after a five-week stay in Bangladesh. He had resided in a dengue-endemic area during this time. Whilst there, he experienced two episodes of fever over the 14 days prior to his return, one episode lasted overnight and one lasted 24 hours. His family also had this fever. The patient additionally experienced nausea, night sweats, loss of appetite, muscle weakness and fatigue. The patient sought medical advice whilst in Bangladesh and was prescribed doxycycline, which resulted in a temporary improvement of his symptoms. Medical investigations and results from this, however, were not available to the emergency department. The patient's symptoms then subsided several days prior to his return. However, two days after arriving in the United Kingdom, he had a resurgence of fever and developed a widespread, blanching macular rash. He also experienced reduced urine output, night sweats, conjunctivitis and facial erythema. He denied diarrhoea, vomiting, chest pain and abdominal pain.

On initial examination, the patient had a blood pressure of 187/108 mmHg, a temperature of 37.2 °C, a pulse of 71 beats per minute, a respiratory rate of 18 breaths per minute, and a GCS of 15/15. He had been febrile at home. A macular, blanching rash was noted over the limbs, torso and neck and back. Tender lymph nodes were felt behind the ears and the patient felt that his face was painful on palpation. No organomegaly was felt on examination. His past medical history included hypertension and diabetes only. Chest X-ray and bloods were normal (Table 1).

**Table 1:** Patient's blood results. MCV: Mean corpuscular volume; MCH: mean corpuscular hemoglobin; MCHC: mean corpuscular hemoglobin concentration; RDW: red cell distribution width; NRBC: nucleated red blood cell; eGFR: estimated glomerular filtration rate; CRP: C-reactive protein; PT: prothrombin time; APTT: activated partial thromboplastin time

Blood Result	Unit	Reference Range	Day 1	Day 2	Day 3	Day 4
WBC	x10⁹/L	4.0–11.0	4.7	4.2	4.4	5.4
RBC	x10¹²/L	4.0 - 6.0	5.03	5.10	5.28	4.96
Hb	g/L	130.0-180.0	139.0	141.0	146.0	135
Hct	L/L	0.400-0.520	0.398	0.406	0.418	0.394
MCV	fL	80.00-98.00	79.1	79.6	79.20	79.40
MCH	pg	27.0-33.0	27.6	27.6	27.7	27.2
MCHC	g/L	320-365	349	347	349	343
RDW	%	11-15	13.0	13.0	13.0	13.0
Platelets	x10⁹/L	150.0-400.0	201.0	170.0	195	214.0
Neutrophils	x10⁹/L	1.80-7.50	2.29	2.62	2.61	3.25
Lymphocytes	x10⁹/L	1.00-4.00	1.55	0.96	1.26	1.49
Monocytes	x10⁹/L	0.20-1.00	0.49	0.41	0.29	0.39
Eosinophils	x10⁹/L	0.00-0.40	0.31	0.19	0.21	0.25
Basophils	x10⁹/L	0.00-0.10	<0.10	<0.10	<0.10	<0.10
Immature Granulocytes	x10⁹/L	0.00- 0.03	<0.10	<0.10	<0.10	<0.10
NRBC	x10⁹/L	< 0.10	<0.10	<0.10	<0.10	<0.10
Sodium	mmol/L	133-146	139	x	x	x
Potassium	mmol/L	3.5-5.3	3.6	x	x	x
Urea	mmol/L	2.5 - 7.8	5.7	x	x	x
Creatinine	µmol/L	59-104	106	x	x	x
eGFR	mL/min/1.73 m²	> 90	70	x	x	x
CRP	mg/L	0-5	6	x	x	x
PT	seconds	9-13	10.6	x	x	x
APTT	seconds	22-36	21.5	x	x	x

Dengue fever was initially considered because of the biphasic nature of the patient’s symptoms. Empirical doxycycline was commenced to cover alternative tropical infections, including leptospirosis and rickettsial disease, which formed part of the differential diagnosis. Other differentials included malaria, mycoplasma pneumoniae, enterovirus, adenovirus, and measles. A plan was made to escalate antimicrobial therapy to ceftriaxone should the patient’s clinical condition deteriorate; however, this was not required. The patient remained haemodynamically stable throughout admission, with gradual symptomatic improvement. He was admitted for one day only and his initial malaria screen during this time was negative. Following discharge, he re-presented to the same-day emergency care unit on two consecutive days for repeat malaria screening, all of which were negative. In light of these findings, dengue-specific investigations were deemed unnecessary; no thrombocytopenia or leukpenia were seen, inflammatory markers were normal, the patient's symptoms improved and the symptom timing was not consistent with the typical biphasic course of a dengue infection. The patient was therefore discharged with a diagnosis of a self-limiting viral illness associated with rash. Follow-up blood tests were unremarkable, and no further complications or hospital admissions occurred.

## Discussion

This case highlights a diagnostic challenge in both emergency medicine and infectious disease practice. It shows a febrile returning traveller returning from Bangladesh with a presentation that elicits concern for dengue. However, the clinical presentation does not quite conform to a classic diagnostic pattern. It therefore illustrates how global interconnectivity blurs the boundaries of knowledge between local and tropical infections.

Dengue is one of the most rapidly expanding mosquito-borne viral infections globally. It is transmitted by the Aedes aegypti and Aedes albopictus female mosquitoes. 14.1 million dengue cases and 9508 dengue-related deaths were reported in 2024 [12]. Bangladesh holds one of this region’s largest burdens; in 2023, 1.3 times the number of dengue cases were reported compared to the previous 23 years [13]. Urbanisation in Bangladesh has contributed to the geographical expansion of vector-borne diseases. Climate change further exacerbates this risk; increasing temperatures and worsening humidity amplify mosquito breeding [14].

Dengue typically manifests after an incubation phase of five to seven days and subsequently progresses through the febrile, critical and recovery phases. The febrile phase presents with fever lasting up to seven days and can present biphasically. Myalgia, retro-orbital pain, and a macular or maculopapular rash are also often seen. The critical phase occurs between days three and seven [15]. Temperature decreases and elevated aspartate aminotransferase, thrombocytopenia and leukopenia are seen [16]. The recovery phase lasts around three days [15]. Dengue confirmation varies between facilities globally; however, ideally, a positive nucleic acid amplification test (e.g., reverse transcription polymerase chain reaction) or non-structural protein 1 (NS1) antigen test confirms an acute dengue infection [17]. The NS1 antigen is most sensitive within the first five days of illness [17]. In this case, the prolonged duration of symptoms placed the patient outside the optimal diagnostic window, and laboratory features were not consistent with dengue. While a remote dengue infection followed by a secondary, superimposed viral illness cannot be excluded, active dengue was unlikely and therefore did not warrant dengue-specific testing.

Importantly, the clinical overlap of dengue with other tropical infections including leptospirosis and chikungunya and common infections like COVID and influenza complicates assessment [18]. Shared features such as fever and rash can obscure the diagnosis in returning travellers. In acute settings, rapid viral respiratory swabs are often easily accessible and can both inform and exclude diagnosis as point-of-care testing [19]. Conducting these tests can guide management decisions and provide clinicians with a clearer diagnosis to guide further care. Additionally, this can help minimise unnecessary isolation durations and alleviate patient anxiety.

The case reinforces the importance of obtaining a comprehensive travel history. This will give clinicians a better idea of the diagnosis and the management plan going forward. The travel history should include details such as the destination, particularly the length of time spent there and the date of return. Itinerary (including layovers and flight connections), rural areas visited, prior pre-travel vaccinations, adherence to any chemoprophylaxis and the severity and timing of symptoms are all crucial questions [20]. Epidemiological contexts and symptom timings must therefore be carefully explored and interpreted. Additionally, it is important to assess potential exposure risks, such as animal stings or bites, water sources, and contact with bodily fluids. Even if recent travel is not reported, conditions like malaria and tuberculosis can remain dormant and may manifest later. Therefore, it is advisable to inquire about travel history for all patients and to investigate further when appropriate. 

## Conclusions

Fever in returning travellers necessitates a systematic, balanced approach. Dengue symptoms overlap with many common infections; awareness of presentation, timing, and symptoms is crucial. This does not mean tropical infections, such as dengue, should be overlooked. Instead, it highlights the importance of considering common infections and performing routine investigations. A structured approach including viral testing and detailed travel history ensures accurate diagnosis and management. Ultimately, clinicians should recognise that not all fevers in returning travellers are from travel-acquired infections.
